# The work of case managers as experienced by older persons (75+) with multi-morbidity – a focused ethnography

**DOI:** 10.1186/s12877-015-0172-3

**Published:** 2015-12-17

**Authors:** Markus Hjelm, Göran Holst, Ania Willman, Doris Bohman, Jimmie Kristensson

**Affiliations:** Department of Health, Blekinge Institute of Technology, SE-371 45 Karlskrona, Sweden; Department of Health Sciences, Lund University, SE-221 00 Lund, Sweden; Department of Care Science, Malmö University, SE-211 18 Malmö, Sweden

**Keywords:** Aged, Case management, Comorbidity, Continuity of patient care, Delivery of health care, Ethnography, Intervention, Multi-morbidity, Qualitative research

## Abstract

**Background:**

Complex health systems make it difficult for older persons (75+) with multi-morbidity to achieve continuity of care. Case management could be one way to address this difficulty. Currently, there is a need to extend the knowledge regarding case management as experienced by those utilising the services, namely older persons (75+) with multi-morbidity. The study aimed to explore older persons’ (75+) with multi-morbidity experiences of case managers.

**Methods:**

The study design was qualitative and used a focused ethnographic approach. Data was collected through individual interviews with 13 older persons and by participant observations with accompanying field notes, all conducted in 2012–2013.

**Results:**

The data revealed four themes illustrating the older persons’ experiences of case managers:

1) Someone providing me with a trusting relationship; 2) Someone assisting me; 3) Someone who is on my side; and 4) Someone I do not need at present.

**Conclusions:**

This study illustrates the importance of establishing trusting relationships between older persons and their case managers in order to truly provide assistance. The older persons valued the case managers acting as informed but unbiased facilitators. The findings could be of help in the development of case management interventions better designed for older persons with multi-morbidity.

## Background

Older persons with multi-morbidity i.e. multiple independent diseases, are at risk of receiving fragmented care because of complex health systems [1–5]. One way of addressing this risk could be the use of case management. Case management aims to improve the coordination of health and social care, with the case management interventions being performed by case managers [6]. Previous research regarding case management interventions targeting older persons has displayed mixed results, for example some results have indicated decreasing service use and costs while other results displayed no change at all following the intervention [5, 7, 8]. Furthermore, within these studies, the interventional elements are often described in limited detail, making it difficult to assess what had actually been done as an intervention [2, 5, 9, 10]. There is a need for knowledge regarding experiences of what actually takes place during a case management intervention from the perspective of those receiving the intervention [8], in this case the older persons with multi-morbidity. Knowledge derived from such studies could therefore help us to better understand and further advance the progress and design of case management interventions aimed at older persons with multi-morbidity.

Among Europe’s aging population, a substantial number of older persons have multi-morbidity [11, 12]. In Sweden, having several chronic diseases is considered to be the most common state of health for persons aged 75 years and older [13]. The same is seen internationally, where the prevalence of multi-morbidity varies between 55 to 98 % amongst persons aged 65 years and older [14]. The definition of multi-morbidity set by the Swedish National Board of Health and Welfare in 2003 [15] is: “being over 75 years, having three or more medical diagnoses from different disease groups and also been acutely admitted to hospital at least three times during the last twelve months” [15]. The prevalence according to this definition, a definition also used in the current study, is estimated at 7 % of the Swedish population [13]. Studies suggest that older persons with multi-morbidity can experience difficulty feeling involved in their own care because of poor care coordination and a high waiting time [16, 17]. They are at risk of experiencing difficulties when coordinating care efforts, which could lead to a lack of continuity of care [1, 4]. Lack of coordination and the lack of an individual approach within the health system for older persons exist throughout the Western world [6]. This unsatisfactory coordination could also put older persons’ health at risk, as they might not receive the help they require [18]. This may also have implications for older persons’ well-being as they risk lacking energy to cope with other activities that provide meaningfulness to their lives [4]. To reduce the fragmentation of care, it has become increasingly important to develop, evaluate and gain knowledge about different models aimed at improving continuity of care [2], with one such example being case management.

**Fig. 1:**
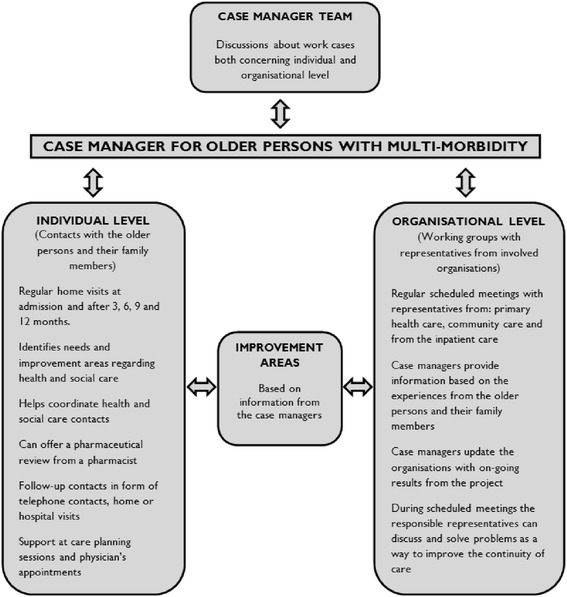
Design of the Blekinge case management intervention. Reprinted from “Case managers for older persons with multi-morbidity and their everyday work – A focused ethnography.” by Gustafsson et al. BMC Health Serv Res. 2013;13:496. Reprinted with permission

Various forms of case management have been tested in order to improve the care process for older persons with multi-morbidity. These case management interventions range from targeting purely financial matters to applying a more individualised approach addressing the older person’s perceived needs [6, 19–22]. Furthermore, these case management interventions range in intensity of provided case management services as well if the services are provided directly by the case managers or instead being coordinated so that other health and social providers perform needed services [6, 19–22]. The Case Management Society of America has defined case management as “a collaborative process of assessment, planning, facilitation, care coordination, evaluation, and advocacy for options and services to meet an individual’s and family’s comprehensive health needs through communication and available resources to promote quality cost-effective outcomes” [23]. These different collaborative processes aim to provide better integrated care for older individuals [24]. The current case management intervention, here further described in the context section, has the purpose of improving continuity of care for older persons (75+) with multi-morbidity. The case managers, within this intervention, utilised an individualised approach whilst also coordinating services i.e. first identifying the differing health and social care needs amongst the older persons. Afterwards, tasks required for addressing these identified needs were coordinated with other health and social care contacts. Elements of the intervention are further described in Fig. 1. Current case management intervention was instigated as the Swedish government and Swedish Association of Local Authorities and Regions, SALAR, expressed a need to improve continuity of care. Thus, funded interventions with integrated health and social care solutions aimed at older persons with complex health needs. Case management was expressed as one possible way of assisting vulnerable sub-groups such as older persons with multi-morbidity.

Research indicates the importance of case management interventions in improving the continuity of care for older persons with multi-morbidity, but also that case management interventions are often described in limited detail, making it difficult to assess what has actually been done as an intervention [2, 5, 9, 10]. Previously, there have been few studies focusing on older persons’ experiences of case management interventions. According to Sandberg and colleagues [25], the older persons considered their case managers to be an additional resource for help, support and guidance [25]. The importance of support was also identified in a study by Sargent and colleagues [26] where the older persons valued the psychosocial support of the case managers as being equally important to the clinical care. Brown and colleagues [27] reported that older persons appreciated the support, care, and confidence-building that the matrons i.e. nurse case managers provided through their case management services. Similar results were found in a study by Nelson and colleagues [28]. They reported that the relationship between the case manager and the older person was of paramount importance. This relationship enabled the case manager to serve as a liaison between the patient and the physicians assisting the older person in the process of navigating within the health system [28]. However, none of these studies have had a particular focus of older persons with multi-morbidity. Moreover, many aspects, such as context, interpersonal relationships and unknown facets, could be valuable to the success of a case management intervention. Therefore, case management interventions need to be explored in terms of what has been conducted within a specific context. Ethnographic methods would be useful in exploring people’s experiences of case management interventions since ethnographic methods emphasise an in-depth understanding of the specific context in which the experiences occur. Furthermore, ethnographic methods could facilitate a deeper understanding of case management by taking into account multiple perspectives i.e. the older person’s perspective as well as the researchers own perspective of the case management intervention [29]. The current case management intervention (see Fig. 1) was conducted in the context of the Swedish health system, targeting a population of older persons (75+) with multi-morbidity. Knowledge derived from this study could help us to gain deeper knowledge regarding case management and so be of help in the improvement of case management interventions. Consequently, the aim of this study was to explore older persons’ (75+) with multi-morbidity experiences of case managers.

## Methods

### Design

The study design was based on the approach of focused ethnography [29], situated within interpretive ethnography [30, 31]. Data collection consisted of individual interviews with 13 older persons as well as participant observations comprising field notes, all conducted in 2012–2013. Focused ethnography is appropriate for exploratory research focusing on a subgroup sharing a common experience [29], such as older persons with multi-morbidity and their experiences of case managers. Focused ethnography allows for the exploration of experiences from both an “emic” perspective i.e. an inside perspective as experienced by the older persons and an “etic” perspective i.e. an outside perspective [32] as experienced by the researcher during participant observations.

### Setting

During the years 2011 to 2013, there was a case management intervention in Blekinge County in Sweden (see Fig. 1) with the purpose of improving continuity of care for older persons (75+) with multi-morbidity. Blekinge is a county in the southern part of Sweden, populated by approximately 150,000 inhabitants, and consists of both rural and urban areas. Blekinge has one regional hospital and one local hospital serving its inhabitants. The health system consists of primary health care, community care and inpatient care. The County Councils are the regional providers of health care and the five municipalities are mainly responsible for the care of older persons living in nursing homes or needing home services. Prior to this study, the health system was unaccustomed to case management services for older persons with multi-morbidity.

The case management intervention was based on case managers being individual contacts for older persons (75+) with multi-morbidity (see Fig. 1). A total of ten case managers were active during the intervention, of whom three case managers quit at some stage during the intervention. Their different professional backgrounds consisted of: nurse managers (*n* = 3), registered nurses (*n* = 3), assistant nurses (*n* = 3) and one occupational therapist (*n* = 1). The reason behind utilizing case managers with different professional background originated from the importance of having a team that could provide different knowledge and perspectives, being familiar with different parts of the health system. More detailed information regarding the characteristics of the case managers has been provided in a previously published study [33]. The case management intervention consisted of activities performed at two levels (see Fig. 1). The first such level was the individual level, where the case managers identified various changing needs amongst the older persons and their family members and coordinated other health and social care contacts to perform the required tasks. The second level was the organisational level, where the case managers identified and reported deficiencies within the continuity of care to the relevant representatives. One of the case managers’ main functions was thus to provide coordinative services whilst also monitoring the older persons in various processes within the health system. Thus, aim to improve the continuity of care for older persons with multi-morbidity and their family members. The older persons were not actively participating at the organisational level, only at the individual level. The intervention was not linked to any current health or social care organisation and was located within a new and only temporary organisation.

### Recruitment and participants

Recruitment was performed by purposive sampling [34]. The participants were recruited from the intervention project described above [33]. The inclusion criteria were based on the definition of multi-morbidity set by the Swedish National Board of Health and Welfare [15] i.e. over 75 years, three or more medical diagnoses from different disease groups and who also have been acutely admitted to hospital at least three times during the last twelve months. The participants must have been involved in the intervention for at least nine months, to ensure that they had experienced most parts of the intervention.

We were also interested in recruiting those participants who had utilised the case managers’ services the most extensively, with the assumption that these persons could provide us with richer and more detailed narrations. Therefore, the case managers themselves assisted with recruiting participants, as they were knowledgeable about those participants. They informed potential participants both verbally and in writing about the purpose of the study. Potential participants who showed an interest was subsequently contacted by the first author (MH) and given further information about the study. None declined to participate. The participants decided a time and venue for the interview.

### Data collection

Participant observations with a focus on the case managers’ everyday work were conducted in 2012 as part of a larger study [33]. These observations consisted of 125 h with accompanying field notes. Out of these 125 h, 26 h involving 17 observations were conducted during the case managers’ face-to-face interactions with older persons with multi-morbidity. This observational data comprising field notes described the etic perspective [32] i.e. the researcher’s perspective. By utilising this observational data in the current study, the researchers could return to the field that had been studied beforehand, thus gaining a deeper understanding of the context and of the older persons’ experiences. This observational data was intended to be present as a pre-understanding during the data analysis whilst also utilized to compare findings originating from the interview data.

Individual interviews comprising the emic perspective [32] i.e. the participants’ perspectives were conducted by first author (MH) with 13 older persons in 2013 (see Table 1). A semi-structured interview guide [35] was used to guide the interviews, and the participants were encouraged to speak freely around the questions. The semi-structured interview guide contained questions such as: What were your first thoughts when the case manager contacted you? Tell me, what do you and your case manager talk about? Can you tell me about any situations where the case manager has assisted you in any way? Probing questions were used to further deepen the interview. The interview sessions were recorded using a digital voice recorder and then transcribed verbatim to text. All interviews were conducted in the participants’ homes. The interview sessions lasted around 40 min.

**Table 1:** Demographic characteristics of the participants (*n* = 13)

Age (year)	
Mean	82
Range	77–88
Gender	
Women	10
Men	3
Education	
Primary level	9
Secondary level	3
University level	1
Marital status	
Married	2
Widow/widower	9
Divorced	2
Living conditions	
Own housing	13
Home health care services	
Yes	8
No	5

### Data analysis

Data analysis was inspired by Roper and Shapira’s framework for ethnographic analysis [36]. The purpose of this analysis was to describe the participants’ shared experiences. As an on-going and interwoven procedure during data analysis, field notes from the participant observations [33] were read and reflected upon. This was done in order to gain a deeper understanding of the older persons’ experiences of case managers from an etic perspective. This formed a pre-understanding that was constantly present during the data analysis’ different strategies. This pre-understanding aimed to improve the quality of the data analysis. Roper and Shapira’s framework involves five strategies, all of which were used:

- *Coding for descriptive labels*. The interview material was grouped into descriptive labels and then organised in order to compare, contrast and identify patterns.
- *Sorting for patterns*. These descriptive labels were then used to explore potential connections within the material, after which themes were created.
- *Identifying outliers*. Situations were identified that did not match the rest of the findings. These situations were used to critically reflect on the data in order to gain a deeper understanding.
- *Generalising constructs and theories*. In the discussion, the findings were related to other literature and research as a way of discussing and enriching the findings.
- *Memoing with reflective remarks*. Memos were written throughout the research process containing different insights regarding the data. These memos helped to keep track of assumptions throughout the research process.

During the course of data analysis, we moved between the different strategies, going from whole to parts, in order to gain a deeper understanding of the data. Reflective meetings were also regularly held and involved all the authors critically discussing the data analysis and its findings. This iterative process resulted in four themes describing how older persons (75+) with multi-morbidity experienced case managers. As a finalizing step observational data was compared with the content of the four emerging themes, integrating an etic perspective of the findings.

### Ethical considerations

This study was conducted in compliance with the established ethical guidelines of the Declaration of Helsinki [37]. All participants received both written and verbal information concerning the study, its purpose, and information about how the data would be treated confidentially. Written informed consents were retrieved from all of the participants. Ethical approval was sought and received from the Regional Ethical Review Board in Lund (Dnr 2012/228).

## Results

The findings originate from the ethnographic analysis describing how older persons (75+) with multi-morbidity experienced case managers. From the data, four themes emerged: 1) Someone providing me with a trusting relationship; 2) Someone assisting me; 3) Someone who is on my side; and 4) Someone I do not need at present.

### Someone providing me with a trusting relationship

Most of the participants stated that they experienced trusting relationships with their case manager. The participants felt a genuine trust in the case managers’ abilities and intentions to help them, and stated that the case managers had been available for them during times of need. They trusted that the case managers would always try their best in order to help them with their current concerns. Their trusting relationships with the case managers made them feel more secure that they would cope if future concerns related to their health and social care were to arise:

“*If you need help they can assist you … that’s the advantage, it gives you a sense of security, you know that there is somebody who can help you.*” (Participant 2)

The participants perceived that the case managers were fit for their jobs and they also noticed that the case managers appreciated their current assignments. They expressed a liking for the case managers’ personal traits. Some of the common personal traits associated with the case managers were: easy to talk to, friendly, light-hearted, cheerful and sensible. These traits were considered beneficial amongst the participants in order for them to open up and start trusting the case managers:

“*You have to ‘read’ a person and … I found that she was nice, which makes you feel that you can open up.*” (Participant 3)

The participants spoke about how the case managers engaged in social conversations with them, which could be about everyday things. They appreciated that the case managers took time to sit down and listen to parts of their life history. Some of the participants noted the contrast between these conversations and their former experiences of a lack of time for conversation when interacting with staff from the health system:

“*Really good access … considering that nowadays health care has gone wrong and professionals don’t have time to do anything, but she has time and you can really speak your mind.*” (Participant 3)

The participants expressed how their trusting relationships with the case managers resulted in the possibility to reflect on their current concerns. One of the participants expressed it as the case managers being able to provide them with a different viewpoint regarding their concerns. Thus, they were able to widen their own perspectives concerning parts of the health system:

“*It’s always good to talk to somebody who can take a broader perspective than I can as I’m in the middle of it. I’m sort of unable to see the whole.*” (Participant 13)

For some of the participants, these trusting relationships meant that they could more honestly communicate about how they felt, in contrast to the communication with their own family members:

“*You dare say how you feel, which you can’t really do with the children, or they don’t understand, don’t take it in, but say … that’s good, you can, you’ll cope, you’ll manage.*” (Participant 3)

During participant observations findings illustrated in the theme were observed. During observations same personal traits being expressed by the participants were observed and also a willingness amongst the case managers to sit down, take their time and talk about everyday life such as family situation, leisure activities or just the weather. During their conversations the case managers often tried to turn negative situations experienced by the participants into more positive situations, whilst trying to motivate the older persons to come up with feasible solutions for their current challenges.

### Someone assisting me

The participants described how the case managers provided assistance to them in numerous ways: by mediating health and social care contacts, investigating their concerns, giving advice, as well as assisting them in making sense of parts of the health system. They commented on how the case managers were readily available and capable of assisting them with their current concerns. They spoke about how the case managers assisted them in navigating parts of the health system:

“*She told me where to seek help and where to phone.*” (Participant 11)

They further described how the case managers asked them numerous questions. These questions involved different subjects concerning their current health situation, disease status, current life satisfaction and current medications. They were asked if they needed any assistance as they raised concerns during the questioning. They were also asked by the case managers how they perceived their current contacts with the health and social care service providers:

“*Yes, and then she wants to know what I think and if I’m satisfied with the service I get.*” (Participant 6)

At times, the participants’ family members were present during their interactions with the case manager. They believed that their family members benefitted from the case managers as they also received information concerning the health system. Many of the participants did not find it difficult to seek assistance from the case managers; instead this was described as being a positive experience:

“*You feel very welcome, I mean … it never feels awkward … no, no, you feel you can turn to her about anything and she has said as much, just phone if you’ve got something on your mind.*” (Participant 3)

The participants were provided with different types, both oral and written, of information. The written information consisted of leaflets containing information on where to seek the required help. They often did not read these leaflets but rather viewed them as being a useful resource in the event that something happened to them. The oral information could consist of any information related to the health system. They described how they believed the case managers to be knowledgeable about different aspects concerning the health system. If there was something they could not understand, for instance if they received a complicated letter, they noted the opportunity to call the case managers for assistance:

“*If there’s something I don’t understand. Because sometimes the written information you get can be a bit complicated. You only understand the half of it.*” (Participant 1)

The participants were assisted by the case managers to mediate between different health and social care contacts. They were often instructed how they themselves should proceed with different tasks in order to get the necessary help, for example receiving contact information from the case managers with which they could contact specific health professionals. At times, the case managers took on more active roles, subsequently performing the necessary tasks for the participants by themselves e.g. aiding the participants by first investigating their current concerns. Afterwards, the case managers reported back to the participants with the updated information:

“*They took samples and suchlike and … a long time passed but I heard nothing, then she phoned and asked them, yes she did. So I finally got an answer.*” (Participant 11)

Findings from the interviews were also presented during participant observations. For instance, the participants expressed being asked a lot of questions by the case managers. Observations showed that the case managers used a questionnaire asking the participants questions regarding their current health situation. The questionnaire was delivered orally and was of help in bringing forth current health needs amongst the participants, as they had to articulate their answers. It was also observed how the case managers by actively listening to the older person’s conversation whilst regularly asking follow-up questions were able to identify health needs.

### Someone who is on my side

The participants stated that the case managers were someone who stood on their side, being their own representative in their different struggles with health and social care representatives. They appreciated the case managers’ abilities to advocate for them at those times they felt mistreated. They expressed appreciation that the case managers clearly understood the importance of their concerns and did not belong to any organisations directly involved in their care. At those times when the participants were dissatisfied with their health and social care services e.g. the community health services provided, they were able to contact the case managers. The participants were then provided with information regarding their legal rights and how they could proceed on the matter. One of the participants described it as getting a honest opinion from the case managers. This was in contrast to the information given to him/her by staff and managers of the municipalities:

“*… all your rights, because they don’t tell you about them. Take the representatives of the municipality, they don’t tell you about your rights. They keep quiet about them. You have to find out yourself.*” (Participant 1)

The participants described how the case managers at times helped them to write reports regarding experienced incongruities in certain health and social care situations. They were also helped by the case managers to follow up on the progress of those reports. The participants stated that they got a more honest opinion from the case managers since they were not seen as being representatives of any health or social care organisations. Instead, they were viewed as being on the participants’ side:

“*She’s on my side and she helps me.*” (Participant 13)

The participants were advised by the case managers of what they perceived to be the best options for them. They sometimes admonished different interventions to the participants e.g. argued with the participants that they really should initiate contact with a health professional. The participants viewed this admonishing as a considerate act on the part of the case managers since they thought the case managers acted in their best interests. The participants also perceived the case managers to emphasise that the final decision should be their own and not anyone else’s:

“*But, as I say, it’s a difficult decision and it’s up to me to decide, that’s what she always tells me*.” (Participant 2)

The participants expressed an appreciation for how the case managers were able to step up and advocate for them when they felt mistreated by health professionals. One participant acknowledged this ability to advocate for him/her:

“*And then I noticed that she was capable, wasn’t afraid, didn’t hesitate to approach anyone. And that’s a really good thing.*” (Participant 10)

The participant observations reflected the interviews and it was observed how the older persons received confirmation by the case managers in a way that they were entitled to express their complaints. Furthermore, they were encouraged to express their complaints to those health and social care representatives involved in their care. A specific situation where the current theme was observed was during a care planning meeting at a hospital ward. An older man was planned to be discharged from the hospital to his apartment where he lived alone. Present at the meeting besides the older man were his three children, one nurse working at the ward, a home care organiser and a case manager. During the meeting it was observed that the persons around the older man was talking about and planning his upcoming home situation whilst more sporadically asking the older man about his views. In this situation the case manager regularly changed the focus of the conversation to the older man, both asking for his personal views on those aspects being discussed, advocating for the older man needs as well as positioning the conversation around the older persons own needs. This strategy was employed throughout the meeting and allowed the older man to be more in focus and to more fully express his opinions concerning his forthcoming home situation.

### Someone I do not need at present

Some of the participants stated that they currently had no use of the services provided by the case managers. They spoke about how they did not have any concerns serious enough to ask the case managers for assistance. They acknowledged that the case managers could be of use in the future if their health status was to worsen. Some of the participants were also content managing their current concerns regarding health and social care by themselves or with the assistance of their relatives:

“*No, I’ve had no need … because I’ve been able to manage many things myself and then my children have done them. So I haven’t needed to ask for her help.*” (Participant 11)

A few of the participants narrated that they did not want to use the case managers’ services as they wanted to manage by themselves, even though they had some concerns. They described it as them being stubborn or that they already knew how they should proceed with their concerns. One of the participants described it as not wanting to become lazy by starting to ask for assistance from the case managers:

“*Of course it’s easier when you don’t have to do it yourself … laughing … Then the laziness in me gains the upper hand. So that may be the case.*” (Participant 8)

Other participants described how they were not in need of the case managers’ assistance since they were currently satisfied with the provided health and social care services and therefore did not know what the case managers could do for them:

“*I have the home help service and they accompany me to the hospital and any health and dental care I need and I don’t need a case manager for other things.*” (Participant 12)

Some of the participants viewed their contact with the case managers as being more of an insurance for the future, in case problems were to arise later on. They expressed an acknowledgement that if they were to be in worse condition later i.e. less clear-headed or having no relatives taking care of them, the case managers could then be of use to them:

“*It could be something that you might need later on. We’re aware that we know nothing about tomorrow, what it’ll be like.*” (Participant 8)

During participant observations the older persons’ willingness to manage by themselves or with the help of their family members was observed. Despite, displaying a willingness to manage by themselves, they also expressed appreciation when being offered assistance by the case managers.

## Discussion

The findings of this study illustrate older persons (75+) experiences of case managers in different ways i.e. someone providing them a trusting relationship, someone assisting them, someone who is on their side, as well as someone who they did not presently need. This knowledge is important in order to gain a deeper understanding of case management interventions. One central finding of this study is the desire among the participants to establish a trusting relationship with the case managers. The participants’ narratives displayed a need for the case managers to support them as individuals, covering their emotional needs. This is in line with Sargent, Pickard, Sheaff and Boaden’s [26] findings, which strongly emphasised the need for implementing psychosocial support into case management models designed for frail older persons. Even though this support of emotional needs was not considered to be an active part of the intervention (see Fig. 1), it still played a major role for the older persons. Similar results signifying the importance of supporting emotional needs amongst older persons have been found in a previous case management study [25]. Furthermore, the older persons’ narratives recognised the case managers as being there for them and aiding them with their individual concerns. Other literature suggests that this kind of person-centred approach supports a respectful person-provider relationship [38, 39]. The narratives also highlighted the importance of the case managers not representing any of the health or social care organisations linked to the older persons’ care. Kane [40] suggests that, for professional guidance to be effective, this guidance might have to be offered by an informed but disinterested facilitator [40]. This view aligns with previous findings from our study exploring the case managers’ perspective. The case mangers expressed that older persons trusted them to speak on their behalf since they did not belong to a profession with organizational affiliation [33].

Experiencing trust in the case managers appeared to be an important facilitator for the older persons to share their life situations with the case managers and enable them to share their concerns. In our previous study [33] the importance of trust was highlighted. The case managers expressed that without this trusting relationship to the older persons they could not perform their tasks [33]. These findings align with other research confirming the prerequisite of trust between case managers and older persons if their needs are to be adequately provided for [10, 25]. Furthermore, our study highlights interpersonal aspects as important facilitators of trust, for example the participants’ connotation of the case managers’ personal traits as well as the case manager providing satisfactory time to listen to the participants’ narrations of everyday things. Another important finding from our study is that trust seems to be important in allowing the information exchange to function well. These findings are in accordance with findings by Robben et al. [41], where aspects such as having enough time and establishing good relationships were found to be crucial in providing a satisfying information exchange [41].

Some of the participants stated that the case managers were not of use to them at present. Despite our inclusion criteria targeting those participants utilising the case managers’ services most extensively, this was still apparent in our findings. Participant observations provided us with more insight regarding the participants’ experiences. It was observed that the current theme could be an expression from the participants in wanting to manage by themselves. Another possibility could be the circumstance that the participants actually have forgotten receiving assistance from the case managers, in relation to the intervention spanning over a longer period of time. These findings illustrate that we cannot evaluate case management interventions from just one perspective, but instead need to look at the experiences of case management interventions from multiple perspectives to get a fuller picture. This could also highlight the difficulty of finding those persons in most need of case management. One of the core concepts in case management is case finding [42]. Case management as an intervention aims to be cost-effective, and it is of utmost importance that resources target those individuals in greatest need [42]. Current case management intervention utilized the inclusion criteria of multi-morbidity set by the Swedish National Board of Health and Welfare [15]. Findings from current study and our earlier ethnographic study [33] indicates that for most of the participants the case management intervention was of great importance to them. However, findings also indicate that some of the participants involved might not be those older persons in greatest need of case management. Previous research, display that it is a real challenge to define which target group is most fitting for this type of intervention [5, 7, 8]. It seems that we cannot rely on just one definition of a target group, but also have to keep the possibilities open for recruitment of additional older persons seeking case managers’ assistance. Due to the complexity of needs amongst older persons with multi-morbidity as well the importance of interpersonal aspects, the recruitment process might have to be kept more open. There is also a need for additional knowledge regarding which older persons are at risk of needing help in the future so that recruitment for case management interventions can be pro-actively conducted.

### Methodological considerations

Focused ethnography was used, including individual interviews and participant observations with field notes, to explore the shared experiences of case managers of persons belonging to a specific subgroup i.e. the older persons (75+) with multi-morbidity. Since the design of this study is qualitative, we have chosen to discuss the methodological considerations in regard to the concept of trustworthiness. Trustworthiness is a set of criteria for scientific rigour within qualitative research, as proposed by Lincoln and Guba [43], and consists of transferability, credibility, dependability and confirmability. Transferability is the degree to which the findings can apply or transfer beyond the current study. Credibility is an evaluation of whether or not the findings represent a credible interpretation of the data drawn from the participants’ original data. Dependability is a valuation of the quality of the processes of data collection, data analysis, and theory generation. Confirmability measures how well the findings are supported by the data [43].

In this study, a sampling technique named purposeful sampling [34] was used in order to recruit those participants with the most extensive experience of case managers. According to Malterud [44], choosing a sampling technique can be one way to enhance the credibility of a study. It is important to have a clear description of how and why the sampling was conducted. By using sampling techniques, the relevance can also be increased. This can also better clarify the transferability of this study since the sample has been more clearly defined for the reader [44]. However, one methodological consideration affecting the transferability of the results was the fact that the sample was unevenly distributed amongst gender, since three men and ten women were interviewed.

The lengths of the interviews were moderate and, even though the participants mostly spoke about their experiences of case managers, they expressed a forgetfulness regarding parts of their experiences. This forgetfulness might have resulted in less rich interviews. This might indicate that a more suitable method of interviewing would be through repeated interviews, to obtain richer data. Repeated interviews could also enhance the dependability [43] of this study since changes occurring over time may affect the findings. By conducting repeated interviews, this ever-changing context could have been better accounted for.

One limitation of the study was related to the recruitment process. The case managers may have chosen to inform us about those participants they believed would give us the most positive view of them. However, we wanted to find those participants with an extensive experience of case managers and this meant we had to go to the case managers directly, as they were the only ones knowledgeable regarding these potential participants. However, our findings display how some of the participants expressed no need of the case managers’ services, indicating that not only positive aspects were put forth during the interviews.

A methodological strength of this study is that the systematic steps of the ethnographic analysis are clearly described. Also, to enhance the credibility of the findings, various quotations from the texts of the interviews were used in the results. Another strength of this study’s credibility and confirmability was the circumstance that all of the authors participated in the analysis processes i.e. conducting triangulation [45]. Triangulation was also performed as the first author had conducted participant observations with accompanying field notes [33]. This observational data originating from an etic perspective was used by the authors to return to the field of study in order to gain a deeper understanding of the older persons’ experiences of case managers. According to Flick [45], triangulation in ethnographic research can help to disclose different viewpoints on specific issues and endorse the quality of the research within ethnographic research.

## Conclusions

The findings from this study illustrate the importance of establishing trust between the older persons and their case managers in order for them to be able to truly provide assistance. There should be active elements in case management interventions specifically facilitating trust at the beginning of the admission of participants. Furthermore, as illustrated in the findings, some of the participants did not have any current need of case managers. For these participants, it might be enough to just establish contact as a way of intervening proactively if problems were to arise later on. Another important conclusion is that, even though the participants acknowledged the worth of the active elements of the case management intervention: identifying needs, assessing needs, planning and coordinating health and social care services, they actually highlighted the support of their emotional needs. These findings indicate a different focus of the participants’ individual needs with regard to the interventional parts. This importance of supporting emotional needs is something that should also be considered in the general care of older persons with multi-morbidity. These findings could be of help in the development of case management interventions better designed for older persons with multi-morbidity.

## Abbreviation

SALAR: Swedish Association of Local Authorities and Regions
